# The first‐generation phosphodiesterase 5 inhibitors and their pharmacokinetic issue

**DOI:** 10.1111/andr.12683

**Published:** 2019-07-26

**Authors:** A. Zucchi, E. Costantini, F. I. Scroppo, M. Silvani, Z. Kopa, E. Illiano, M. G. Petrillo, L. Cari, G. Nocentini

**Affiliations:** ^1^ Department of Surgical and Biomedical Sciences, Urology and Andrology Clinic University of Perugia Perugia Italy; ^2^ Andrology and Urogynecological Clinic Santa Maria Hospital University of Perugia Perugia Italy; ^3^ Urology Unit Ospedale di Circolo di Varese Varese Italy; ^4^ Urology Department Santa Rita Clinic Vercelli Italy; ^5^ Andrology Centre Department of Urology Semmelweis University Budapest Hungary; ^6^ Signal Transduction Laboratory Department of Health and Human Services NIEHS, NIH Durham NC USA; ^7^ Department of Medicine Section of Pharmacology University of Perugia Perugia Italy

**Keywords:** erectile dysfunction, excipients, ODF formulation, OTF formulation, PDE5 inhibitors, pharmacokinetic

## Abstract

**Background:**

Erectile dysfunction (ED) is a relatively frequent disease that negatively impacts the overall quality of life, well‐being, and relationships. Although the use of phosphodiesterase 5 inhibitors (PDE5is) has revolutionized the treatment of ED, a high percentage of ED patients discontinue PDE5i treatment.

**Objectives:**

(i) To analyze the reasons for patient dissatisfaction leading to PDE5i discontinuation; (ii) analyze the pharmacokinetics of new formulations focusing on the time needed to reach an effective plasma concentration of PDE5is (T_onset_) following drug intake; and (iii) summarize the physicochemical properties of sildenafil to understand which excipients may increase the absorption rate.

**Material and methods:**

An online PubMed literature search was conducted to identify English language publications from inception to January 2019.

**Results:**

The main reasons for patient dissatisfaction when using PDE5is on demand are the relatively long T_onset_ after taking vardenafil and sildenafil, including formulations such as film‐coated tablets, fine granules, orally disintegrating tablets (ODTs), and oral thin films (ODFs). The relatively long T_onset_, further worsened when accompanied by eating, highlights the following: (i) the need for planning intercourse, determining partner‐related issues; (ii) issues when having sex before the maximum effect of the drug; and (iii) lower drug‐related placebo effects. Some data suggest that sildenafil is a ‘difficult’ molecule, but T_onset_ can be improved following absorption by buccal mucosa using appropriate excipients.

**Conclusions:**

We conclude that several ODT and ODF formulations can improve the ‘discretion’ issue because they are taken without water, but they have similar pharmacokinetics to corresponding film‐coated tablet formulations. One ODF formulation of sildenafil was characterized by a shorter T_onset_ and could potentially increase patient satisfaction following treatment. However, more clinical studies are needed to confirm the findings. Surfactants and ascorbic acid appear to be crucial excipients for achieving a high absorption rate, but more studies are needed.

## Introduction

Erectile dysfunction (ED) is a disease affecting a relevant part of the male population and increasing with aging. The prevalence depends on the studied population and on the way by which ED is measured. Approximately half of the men aged 40–70 experience some degree of ED (Feldman *et al*., 1994). Among German men aged 30–80, the prevalence of ED was estimated to be 19.2%, with an age‐related increase from ~2% to ~54% (Braun *et al*., 2000). In a Japanese population with a mean age of 56 years, 34% men had severe/moderate ED, and 55% had a mild ED, according to the five‐item version of the International Index of Erectile Function (IIEF‐5) (Imai *et al*., 2010). In an American population with a mean age of 60 years, the prevalence of ED without benign prostatic hyperplasia was ~25%, and in a population with a mean age of 68 years, the prevalence of ED associated with benign prostatic hyperplasia was ~5% (Foster *et al*., 2013). Thus, about one‐third of subjects in their 60s suffers from ED. A recent study shows that only 10% of 70‐year‐old Swedish men declares to suffer from impotence, but 20% of the men did not respond to the survey and several subjects were not sexually active (Stranne *et al*., 2019).

ED is a dysfunction affecting patients even younger than 50. The prevalence of self‐declared impotence in 45‐year‐old men is 1.1% (Stranne *et al*., 2019), but almost 50% of young men complaining of ED are affected by severe ED (Capogrosso *et al*., 2013).

ED has a negative impact on the overall quality of life, well‐being, and relationships (Boyle *et al*., 2003; Althof *et al*., 2006). Indeed, 80% of men and 60% of women aged 40–80 feel that sex is an important part of their lives. A recent study performed on subjects with age > 50 (mean age ~65) reported that men and women who reported any sexual activity in the past year had significantly greater enjoyment of life compared with those who were not sexually active, and among sexually active men, sex more than twice a month was associated with greater enjoyment of life (Smith *et al*., 2019). Therefore, the effects of ED on both the patient and their partner can be devastating (Jønler *et al*., 1995; Fugl‐Meyer *et al*., 1997).

In the past 20 years, the use of phosphodiesterase 5 inhibitors (PDE5is) has revolutionized the classification and treatment of ED, and this first‐line therapy is recommended by the guidelines of all major scientific societies (Bella *et al*., 2015; Hatzimouratidis & Giuliano, 2018). Indeed, several patients suffering from ED with psychological etiology show a significant improvement of the erectile function, irrespective of the presence of premature ejaculation or low desire (el‐Sakka, 2006). Other patients suffer from ED with organic etiology, associated with medical comorbidities. For example, diabetes is one of the most important risk factors of ED, and a strong link can be observed between the severity of erectile dysfunction and the duration of the diseases. Despite duration of diabetes, poor metabolic control and diabetic complications negatively affect the efficacy of PDE5is, decreasing the patient satisfaction, several patients respond to the treatment (El‐Sakka, 2004). Moreover, long‐term PDE5i treatment reduces the flow‐mediated dilation (a physiologic measure of endothelial reactivity) and pro‐inflammatory cytokine levels, which can result in the prevention of endothelial dysfunction (Santi *et al*., 2015).

Since clearance of sildenafil by the Food and Drug Administration (FDA) and European Medicines Agency (EMA) in 1998, new PDE5i compounds have become available, including vardenafil (2003), tadalafil (2003), and avanafil (2013), the latter being a second‐generation PDE5is as compared with first‐generation drugs (sildenafil, vardenafil, and tadalafil). The main properties of PDE5is are reported in Table 1, for which it can be deduced that classification in the first generation and second generation is mainly based on historical reasons. This review focuses on the first‐generation PDE5is.

**Table 1:** Main properties of PDE5is^a^

	Sildenafil (film‐coated tables)	Vardenafil (film‐coated tables)	Tadalafil (film‐coated tables)	Avanafil (film‐coated tables)
Tmax (min), median	45–60 min^b^	45–60 min^c^	120 min	30–45 min
Effect of food on Tmax (min)	Mean delay in T_max_ of about 60 min (high‐fat meal)	None (low‐fat meal) mean delay in T_max_ of about 60 min (high‐fat meal)	None	Mean delay in T_max_ of about 75 min (high‐fat meal)
T1/2 (h)	3–5 h	4–5 h	17.5 h	5–10 h
Metabolism	Hepatic (primarily by the CYP3A4 and to a minor extent, by the CYP2C9)	Hepatic (primarily by the CYP3A4 with contribution from the CYP3A5 and CYP2C)	Hepatic (primarily by the CYP3A4)	Hepatic (CYP3A4, principal route, and CYP2C9, secondary route)
Main PDE targeted	PDE5	PDE5	PDE5	PDE5
Secondary PDE target	PDE6 (10‐times lower specificity)	PDE6 (15‐times lower specificity)	PDE11A (controversial)	PDE6 (100‐times lower specificity)
Very common adverse effects (>10%)	Headache	Headache	None	None
Common adverse effects (<10%, >1%)	Dizziness Abnormal vision Flushing Nasal congestion Nausea Dyspepsia	Dizziness Flushing Nasal congestion Dyspepsia	Headache Flushing Nasal congestion Dyspepsia Back pain Myalgia Pain in extremity	Headache Flushing Nasal congestion

a Data obtained from DrugBank (if not otherwise specified) queried on May 2019 and available at the URL: https://www.drugbank.ca/

b Damle *et al*. (2014), Zheng & Kim (2014), Roh *et al*. (2013), Aguirre *et al*. (2019).

c Heinig *et al*. (2011).

## Search Strategy

An online PubMed literature search was conducted to identify English language publications from inception to January 2019 using combinations of the terms erectile dysfunction, phosphodiesterase type 5 inhibitor, phosphodiesterase 5 inhibitor, PDE5 inhibitor, clinical trial, sildenafil, vardenafil, tadalafil, avanafil, head‐to‐head, unmet needs, patient expectations, patient satisfaction, patient discontinuation, compliance, adherence, solubility, excipients, drug formulations, drug delivery, buccal mucosa, orally dispersible, orally disintegrating, orodispersible, ODT, ODF, and MeSH terms ‘Erectile Dysfunction’ (MeSH Unique ID: D007172), ‘Phosphodiesterase 5 Inhibitors’ (MeSH Unique ID: D058986), ‘clinical trials as topic’ (MeSH Unique ID: D002986), ‘Sildenafil Citrate’ (MeSH Unique ID: D000068677), ‘Vardenafil Dihydrochloride’ (MeSH Unique ID: D000069058), ‘Tadalafil’ (MeSH Unique ID: D000068581), ‘Avanafil’ (MeSH Unique ID: C553414), ‘Patient Satisfaction’ (MeSH Unique ID: D017060), ‘Patient Compliance’ (MeSH Unique ID: D010349), ‘Treatment Adherence and Compliance’ (MeSH Unique ID: D000074822), ‘Solubility’ (MeSH Unique ID: D012995), ‘Excipients’ (MeSH Unique ID: D005079), ‘Drug Compounding’ (MeSH Unique ID: D004339), ‘Drug Delivery Systems’ (MeSH Unique ID: D016503), and ‘Mouth Mucosa’ (MeSH Unique ID: D009061). Other relevant articles were identified by manually reviewing the reference lists of selected articles. Only studies that we considered to be relevant to the pharmacokinetic issue of PDE5is were presented and discussed. Therefore, a limit of our paper is that it is not a ‘systematic review’ and, for this reason, is not eligible for inclusion in PROSPERO.

## The First Generation and the Second Generation of PDE5is

The efficacy of PDE5is has been thoroughly assessed, and all act in a dose‐dependent manner. They potentiate erectogenic signals by increasing the levels of cGMP, the main mediator of erection, through inhibition of phosphodiesterase enzymes, which are divided into 11 subfamilies. PDE5 is the predominant member present in the corpora cavernosa, and sildenafil, vardenafil, tadalafil, and avanafil mainly target PDE5, although all PDE5is exert some inhibitory activities against other PDEs at clinical doses, and this may contribute to both efficacy and adverse events. Thus, the mechanisms of action of different PDE5i compounds are similar but not identical (Kayık *et al*., 2017; Scaglione *et al*., 2017). However, the difference between first generation and second generation of PDE5is is not based on a difference in their specificity (see Table 1).

Adverse side events following PDE5i intake are experienced by a small number of patients. Some adverse events, including headache, flushing, and nasal congestion/rhinitis, are observed at a similar rate following treatment with sildenafil, vardenafil, tadalafil, and avanafil. Conversely, other adverse effects are more peculiar to a particular PDE5i; dizziness is peculiar to sildenafil and vardenafil, alteration in color vision is peculiar to sildenafil, and myalgia and back pain are peculiar to tadalafil. Moreover, dyspepsia is observed at a lower rate after avanafil administration, as compared to the other PDE5is. Thus, even in this aspect, there is not a clear‐cut difference between first generation and second generation of PDE5is (Table 1). All type A adverse effects of PDE5is are dose‐dependent; the higher the dose, the higher the frequency/relevance of adverse effects (as with efficacy). Thus, the optimal dose for each patient must be chosen to achieve the intended effects with minimal adverse events.

The pharmacokinetic properties of the first‐generation PDE5is will be presented in detail in the following paragraphs. T_max_ of avanafil is slightly shorter than those of the first‐generation PDE5is (see Table 1). Moreover, similarly to sildenafil, T_max_ of film‐coated tablets is heavily influenced by food intake, and as discussed in details in the following paragraphs, the majority of the sexual acts takes place after dinner. In this frequent case, the small pharmacokinetic advantage of avanafil as compared to the other PDE5is is completely lost. If the shorter T_max_ of avanafil solves the T_max_/T_onset_ issue of PDE5is, it has to be determined with head‐to‐head studies in the real‐life.

Data from head‐to‐head clinical trials of PDE5is are based on the subjective evaluation of patients and have design flaws and/or brief follow‐up (Govier *et al*., 2003; Ströberg *et al*., 2003; von Keitz *et al*., 2004; Doggrell, 2007; Mirone *et al*., 2009; Raheem & Kell, 2009; Smith *et al*., 2013). One relevant problem in evaluating data from comparative studies is the dose used. In fact, PDE5i agents have different potency, and there is no agreement on the equivalent dose for the correct comparison of efficacy. A meta‐analysis of several studies recently evaluated the efficacy (on 47,626 patients) and adverse events (on 20,325 patients) of PDE5is. Sildenafil at a dose of 50 mg achieves the highest efficacy, but also the highest rate of adverse events (Chen *et al*., 2015). Conversely, 10 mg of tadalafil exerts the lowest rate of adverse events. In conclusion, the authors suggest starting the treatment of ED patients with sildenafil at 50 mg, in particular for patients prioritizing efficacy. On the contrary, the meta‐analysis of Corona *et al*., (2016a) suggests that avanafil has comparable efficacy, but lower incidence of drug‐related side effects, compared to first‐generation PDE5is. More studies and, particularly, head‐to‐head studies will determine which is the best PDE5i, if any.

Our review focuses on the first‐generation PDE5is and in particular on the drugs taken on the on‐need basis (sildenafil and vardenafil), for some reasons: (i) Presently, they are more used than avanafil; (ii) more clinical and pharmacological studies are available; (iii) new formulations of sildenafil and vardenafil have been prepared, raising the possibility that T_max_ and T_onset_ change, in fasting patients and in patients that had a meal.

## Choosing Sildenafil/Vardenafil vs. Tadalafil based on Pharmacokinetic Properties

Most PDE5i formulations on the market consist of film‐coated tablets to be taken orally and absorbed via the gastrointestinal route. In a fasting man, sildenafil is relatively rapidly absorbed after oral intake, reaching the maximum plasma concentration between 30 min and 2 h (median T_max_ = 0.8–1 h) (Hong *et al*., 2017; Hatzimouratidis & Giuliano, 2018). Importantly, a fatty meal delays absorption, increasing T_max_ by ~1 h. Data after a ‘normal’ meal are not available, but we can assume that in a non‐fasting man T_max_ is likely to be between 1 and 2 h. Such conditions are not rare, given that sexual activity peaks in the evening (more than 50% of intercourse events occur between 9 pm and 12 pm (Glina *et al*., 2006)), reasonably soon after dinner. Bioavailability of sildenafil in fasting patients is ~40% because it is extensively metabolized by cytochrome P450 enzymes (mainly CYP3A4), with a half‐life of ~3 h.

The pharmacokinetic properties of vardenafil are quite similar to those of sildenafil. In a fasting man, vardenafil is relatively rapidly absorbed after oral intake, reaching T_max_ after 30 min to 2 h (median T_max_ = 0.9 h) (Klotz *et al*., 2001; Hatzimouratidis & Giuliano, 2018). Bioavailability in fasting patients is ~15%, and the half‐life is ~4 h. Again, a fatty meal causes a decrease in bioavailability.

In a fasting man, after oral intake, tadalafil is absorbed more slowly than sildenafil and vardenafil, reaching T_max_ after 30 min to 6 h (median T_max_ = 2 h) (Klotz *et al*., 2001; Hatzimouratidis & Giuliano, 2018). Absorption and bioavailability are independent of eating, and the half‐life is ~18 h.

**Figure 1:**
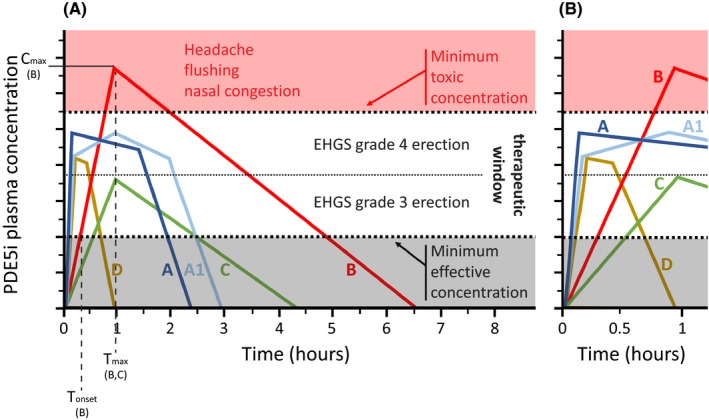
Advantages and disadvantages of plasma concentration curves of PDE5is taken on demand. (A) Schematic diagram of examples of plasma concentration curves that might be obtained with different formulations (A, A1, B, C, and D curves of PDE5is). (B) Magnification of the curves depicted in A during the first hour. The time needed to reach the minimum effective concentration is T_onset_, the maximum drug plasma concentration is C_max_, and the time at which C_max_ is reached is T_max_ (indicated only for the specified formulations). Desired and unwanted effects of PDE5is are depicted. Within the therapeutic windows, lower and higher PDE5i concentrations were considered to give an EHGS grade 3 erection and grade 4 erection (fully rigid erection), respectively. Figure shows the clinical effects of a PDE5i depend on dose, half‐life, and formulation of a drug, determining different plasma concentration curves. Both A and A1 reach a plasma concentration sufficiently high to give a fully rigid erection (EHGS grade 4) after 5–10 min (despite the different T_max_), and their concentrations are sufficiently high for long enough to allow satisfactory sexual activity. The plasma concentrations of B and C, derived from different doses of the same drug formulations, are both suboptimal for adverse effects or efficacy, respectively. The plasma concentration of formulation D is suboptimal for duration of the wanted effect.

New formulations of sildenafil and vardenafil have been prepared in recent years (see the following paragraphs). Several pharmacokinetic parameters have been investigated including the area under the curve (AUC) giving information on the bioavailability of formulations, the maximum plasma concentration (C_max_), and the time at which C_max_ is reached (T_max_), indicating the time of maximum effect. If the shape of the AUC is similar for different drug formulations and T_max_ is shorter, the absorption rate is higher. However, T_max_ gives only indirect information on the rate of adsorption and may lead to wrong conclusions when comparing AUCs of a different shape. The most interesting information is the time following drug intake at which the drug reaches the minimum effective concentration (T_onset_), and thus begins to cause wanted effects. It is also interesting to know whether drugs reach a too high plasma concentration, possibly explaining the unwanted effects (minimum toxic concentration). Thus, the optimal plasma concentration curve for a PDE5i is curve A in Fig. 1. Curves B and C are suboptimal because they represent a longer time to reach the minimum effective concentration. Formulations yielding A‐type curves have a lower T_max_ than those giving curves B and C. However, formulations giving curve A1 have similar T_max_ values to those giving curves B and C, but are similar to formulations giving A‐type curves regarding the rapidity with which the minimum effective concentration is reached (T_onset_), and should be preferred to formulations giving curves B and C. Finally, the effective concentration should be sufficient to maintain efficacy until needed. Curve D indicates a formulation that is suboptimal from this point of view. Obviously, the above reasoning is theoretical and cannot be directly applied to experimental data for two reasons: (1) Minimum effective and minimum toxic concentrations are different in each patient, depending, for example, on the degree of ED and comorbidities, and (2) the median minimum effective and minimum toxic concentrations are not established. Nonetheless, this reasoning should be considered when comparing different drug formulations.

Based on pharmacokinetic properties, sildenafil and vardenafil are considered the best options for on‐demand use. Tadalafil provides an alternative to on‐demand dosing of sildenafil or vardenafil for couples anticipating frequent and/or spontaneous rather than scheduled sexual activities, with the advantage that dosing and sexual activity no longer need to be temporally linked. However, several patients, and particularly older ones, do not engage in frequent sexual activity, and the use of on‐demand dosing is preferred based on cost–benefit analysis. However, tadalafil is the drug of choice in the chronic treatment of patients who underwent nerve‐sparing prostatectomy during the rehabilitation period (Limoncin *et al*., 2017).

## Discontinuation is Much Higher for PDE5is than other Drug Classes with More Side Effects

Although serious adverse events are extremely rare for PDE5i drugs, and most are bothersome but not damaging to health, particularly when taken on demand, a high percentage of patients discontinue pharmacological treatment. In particular, it has been demonstrated that > 50% of men stop treatment with the first‐generation PDE5is in traditional formulations within a year (Corona *et al*., 2015). The discontinuation rate appears high compared, for example, with antihypertensives (21%) (Cummings *et al*., 1982) and TNF‐α blockers (6%) (Costa *et al*., 2017) for which patients do not experience the short‐term benefits of treatment, unlike with PDE5is.

**Figure 2:**
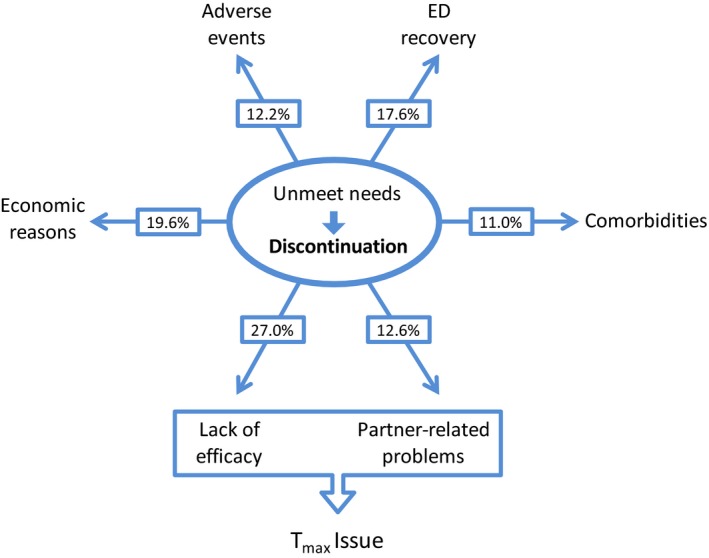
Reasons for the discontinuation of PDE5is according to Corona *et al*. (2016b).

In a study by Corona *et al*., six reasons for discontinuation were described (Corona *et al*., 2016b), as summarized in Fig. 2. Each reason is relevant, as suggested by the fact that no significant difference among factors was detected. As expected, side effects are not the main reason for PDE5i discontinuation, representing just 12% of dropouts, whereas 18% of PDE5i discontinuation was due to ED recovery, related at least in part to the treatment of underlying diseases such as hypogonadism or obesity. A further 20% of PDE5i discontinuation was due to economic reasons, and partner‐related problems accounted for 13% of dropouts. Comorbidities are another reason for discontinuation (11%), possibly representing most of the true non‐responder patients due to heavy damage to the structures responsible for erection.

Lack of efficacy is the last reason with a relevant 27% of the dropouts. Several experts concluded that this reason needs reinterpretation (Sato *et al*., 2007; Carvalheira *et al*., 2012; Corona *et al*., 2016b). Firstly, adequate education on PDE5i use and careful counseling are important factors for increasing the efficacy of the treatment and the success rate (Hackett, 2005; Hatzimouratidis & Hatzichristou, 2007). For example, as explained above, the best time to take sildenafil and vardenafil is 1 h before intercourse if fasting, and 2 h after a meal. Several patients do not follow this advice, as discussed below. Moreover, we should not assume that successful intercourse equates to the full success of the treatment. In fact, about 24% of patients discontinue PDE5i use despite successful intercourse (Kim *et al*., 2014). In general, it appears that dissatisfaction of patients (more than the proportion reporting unsuccessful intercourse) is the main reason for dropout (Sato *et al*., 2007; Carvalheira *et al*., 2012; Jannini *et al*., 2014). In this review, we attempt to understand patient dissatisfaction and focus on the main pharmacokinetics issues of formulations likely responsible. Moreover, we describe and discuss new sildenafil formulations prepared in an attempt to improve patient satisfaction.

## The need for Planning Intercourse in Patients Taking PDE5is Orally

An interesting study by Lopreteet *et al*. on the sexual habits of men with and without ED was performed at the beginning of the PDE5is era when their use was still infrequent (received by only 15% of men with ED) (Eardley *et al*., 2004). All subjects were > 40 years old and heterosexual. The authors found that the median time between thinking about having sex and engaging in foreplay and sex was 5 and 10 min, respectively, and the median time between foreplay and sex was 10 min. Thus, the time elapsed between the first thought about having sex and engaging in foreplay was ~15 min in several men, and the time elapsed between the first thought about having sex and engaging in intercourse was ~30 min. Interestingly, authors did not observe differences in habits between men with and without ED.

Two more recent studies suggest that after 20 years of PDE5is, the habits of PDE5i users have changed. Mulhall *et al*., (2018) interviewed 1458 ED patients from seven countries declaring ED drug use in the prior 3 months. The median age of men was 48 years (interquartile range = 44–55 years). Most men (45%) answered ‘up to several hours in advance’ to the question ‘timing of advanced planning of sexual intercourse’ and only 26% answered, ‘up to 1 h in advance.’ Moreover, most of the interviewed patients strongly agreed (30%) or tended to agree (43%) with the sentence ‘I plan when I am going to have sexual intercourse.’ The change between patients in the first study and those in the present study could be due to the need to take the ED drug at the correct time. Indeed, when men were interviewed about the time to sexual activity commencement (i.e., beginning of foreplay) after PDE5i intake, only 20% answered ≤ 30 min, and 50% answered 30–60 min. Very similar results were obtained when interviewing men with ED from other regions/countries (Jiann *et al*., 2019). Also in this context, no analysis on the comparison between users of specific PDE5i is available.

One reason why ED patients plan sex in advance is that they know, from clinicians or from their own experience, that at least 30 min is needed between drug intake and the desired effects, and usually longer (especially in non‐fasting patients). Thus, the median time elapsed between the first thought about having sex to initiation of foreplay was about 15 min 20 years ago and ~45 min nowadays, at least for ED patients. Although 45 min is sufficient for the effects of the ED drug, this can be too long to wait when both partners are psychologically ready for intercourse. Moreover, if having sex before 30 min after drug intake, the likelihood of a lack of drug efficacy or lack of full efficacy is high. Thus, men know that they must plan and wait before having sex; hence, spontaneity is lost. Moreover, the sex drive of women is not considered under these circumstances. Indeed, men suffering from ED cannot respond promptly to the sexual desires of their partner, even if he takes PDE5i immediately. Moreover, some years ago, <40% of men using PDE5is and suffering from moderate or mild ED shared this information with their partners (Klotz *et al*., 2007), and we can hypothesize that in several instances this caused tension and affected relationships. This might explain, at least in part, the above‐mentioned patient dissatisfaction and partner‐related issues that lead to discontinuation of PDE5i treatment.

In conclusion, the T_max_ issue and the speed with which the drug concentration reaches the optimal therapeutic window (T_onset_) play key roles in the satisfaction of the patients when ED drugs are taken on demand. Rapidly achieving the target plasma concentration (ideally within 15 min) can shorten the time that must pass between PDE5i intake and sexual activity commencement, hereby increasing spontaneity and the satisfaction of both partners.

## The Fine Line between Successful Intercourse and Emotional Well‐Being Following Intercourse

In recent years, several methods to define ED and measure the effects of PDE5is have been used. Interestingly, Montorsi *et al*., (2006) demonstrated in men with ED participating in sildenafil clinical trials that the percentage of erections considered by the patient to be fully rigid, equating to grade 4 of the Erection Hardness Grading Score (EHGS), was positively correlated with sexual satisfaction. Moreover, in this patient group, evaluation of satisfaction at the endpoint of the studies indicated high levels of satisfaction and a positive correlation with erectile function. A similar conclusion was reached by the study of Kaminetsky *et al*., (2009) working on patients treated with sildenafil who showed that satisfaction with the quality of erections correlates positively with changes in the percentage of EHGS grade 4 erections, but not with changes in the percentage of erections that were hard enough for penetration but not completely hard (EHGS grade 3).

Having sex when PDE5i reaches a sufficiently high plasma concentration increases the percentage of erections considered completely hard and fully rigid, and increases sexual satisfaction and well‐being. Either because not instructed correctly by a clinician, or forced by the need to shorten the lag time between drug intake and sex, patients tend to have sex with non‐optimal timing (too soon), hence not receiving the full benefit of the PDE5i effects.

In this context, shorter T_max_ and T_onset_ values (see Fig. 1) may play a role in treatment satisfaction when the drug is taken on demand. In fact, the shorter the T_max_ and the T_onset_ of the drug formulation, the greater the maximum effects anticipated, ensuring the best efficacy during intercourse. In fact, it is reasonable to assume that erections that are hard and fully rigid are reached at the highest plasma concentration of the therapeutic window, and a drug formulation giving a plasma concentration curve of type A and A1 is likely to be correlated with an EHGS grade 4 erection and greater satisfaction. By contrast, a drug formulation giving a plasma concentration C‐type curve has less positive outcomes, and although a higher dose of such curve can achieve an EHGS grade 4 erection (B‐type curve), the risk of side effects is increased.

## Conditioned Responses Favoring A Placebo Effect are Stronger the Shorter the Lag Time between Drug Intake and its Effects

Results from clinical trials demonstrated that the placebo effect is a strong component in response to drugs that increase sexual potency. Interestingly, in routine clinical practice when patients are given a known drug, the effectiveness of the drug is a combination of non‐specific placebo effects and biological effects (Oken, 2008).

Learning and expectations play a key role across all mechanisms responsible for placebo effects. In most experimental protocols studying placebo effects, in order to obtain robust placebo responses, a placebo is given following a preconditioning procedure (Benedetti, 2014). Indeed, several studies suggest an over‐additive interaction between experience and expectancy, most likely due to reinforced expectations (Reicherts *et al*., 2016). After preconditioning, the effects of the drug will be increased by potentiation of placebo effects. It is reasonable to expect that the shorter the lag time between drug intake and its effects, the more efficient the preconditioning, and the stronger the placebo effects. Thus, a shorter T_max_ and T_onset_ may increase the placebo effects of PDE5is and ultimately the effects of the drugs.

## The Need for a Rapid Onset of PDE5i Effects

Jannini *et al*. recently concluded that ‘The ideal therapy aims to be a treatment that responds as much as possible to the normal psychology and naturalness of the relationship’ (Jannini & Droupy, 2019). In this context, particular effort is needed to improve the T_max_ and T_onset_ of PDE5is and increase the speed with which the target plasma concentration is reached. Shortening this lag time likely improves both drug efficacy and treatment satisfaction, as discussed above. Indeed, in a recent survey from the Italian Society of Andrology, two‐thirds of ED patients believe that the most important property of a drug for increasing sexual potency is the rapidity of action (Palmieri *et al*., 2019). A shorter T_max_ might be obtained by new PDE5i molecules, or with new formulations of existing available drugs, improving the velocity of absorption. Meanwhile, considering the high percentage of men that prefer not to share knowledge of ED with their partner, a discreet mode of intake would further improve treatment satisfaction. Avanafil appears to have a shorter T_max_ than sildenafil and vardenafil, but it is still not ideal.

A study performed several years ago by De Siati *et al*., (2003) evaluated the velocity by which sildenafil acted when film‐coated tablets were crushed. The study was performed on 30 patients affected by ED because of vasculopathies or diabetes. For the first 3 months, patients (already treated with film‐coated sildenafil tablets) took the usual dose of film‐coated sildenafil tablets (50 or 100 mg) on demand once a week. They were instructed to take the drug 30 min before planned sexual activity. During the second 3‐month period, patients were instructed to crush the film‐coated tablet in the mouth and leave the crushed tablet under the tongue and in the buccal cavity. They were instructed to take the drug 15 min before planned sexual activity. All patients were asked to annotate the onset of erection, efficacy, and adverse events. The average time needed for full erection for each patient and the mean time needed for full erection with film‐coated tablets and crushed film‐coated tablets were calculated. The authors reported a significant reduction in the initiation of pharmacological activity with crushed film‐coated tablets (29.3 min) compared with intact film‐coated tablets (62.8 min). No differences were noted in the efficacy or the frequency of adverse events. Nevertheless, in four of the 10 patients treated with 100 mg sildenafil, a greater intensity of adverse events was observed with crushed film‐coated tablets. They included flushing, headaches, and nasal congestion.

These results were very interesting, but several flaws affected the study. Firstly, the number of patients studied was low (*n* = 30), even considering the wide age range (45–70 years). Moreover, the treatment was not blinded, and patients were instructed to take the crushed drug 15 min before planned sexual activity (vs. 30 min before planned sexual activity with film‐coated tablets). Although patients were not told about the aim of the study, they could guess that clinicians thought that crushing the tablets may enhance activity based on verbal instructions and common knowledge about a rapid activity of sublingual formulations of other drugs. Therefore, the placebo effect may have influenced the obtained results. Moreover, a granular formulation of sildenafil does not lower the T_max_ but instead increases C_max_ (Zheng & Kim, 2014), which could favor earlier effects. The greater the intensity of adverse events observed with crushed sildenafil at a dose of 100 mg is consistent with this hypothesis.

Despite the flaws, this brief study suggested that the delivery of sildenafil by oral transmucosal absorption may shorten T_max_. Based on this study and the need for a shorter T_onset_, new sildenafil and vardenafil oral formulations have been developed and marketed, as discussed below.

## Oral Transmucosal Drug Delivery Could Potentially Shorten T_max_ and T_onset_ and Improve Treatment Satisfaction

Buccal mucosa is a multilamellar lining consisting of the outer epithelium and basement membrane, supported by the lamina propria and submucosa. The buccal epithelium is a non‐keratinized stratified squamous tissue consisting of ~50 layers of cells, while sublingual tissue contains considerably fewer cell layers (Gandhi & Robinson, 1994). Mucus coats the surface of the epithelium. The area of buccal mucosa is about 100 cm^2^, sufficient to allow drug absorption depending on the physicochemical properties of drugs and excipients.

Several advantages are associated with oral transmucosal drug delivery (OTDD) compared with conventional oral drug delivery, including increased bioavailability and T_max_ and T_onset_ shortening. The absorption of drugs via OTDD appears to occur through two routes: paracellular (between cells), mainly for hydrophilic molecules, and transcellular (across cells), mainly for lipophilic molecules (Zhang & Robinson, 1996).

OTDD can be performed through orally disintegrating tablets (orodispersible tablets, ODTs) and oral thin films (orodispersible films, ODFs). ODTs are defined as a solid dosage form containing medicinal substances that disintegrate rapidly, usually within a matter of seconds, when placed upon the tongue (Center for Drug Evaluation Food Research, 2008). ODFs are defined as thin, flexible, non‐friable polymeric film strips containing one or more dispersed active pharmaceutical ingredients intended to be placed on the tongue for rapid disintegration or dissolution in the saliva prior to swallowing for delivery into the gastrointestinal tract (Kathpalia & Gupte, 2013).

ODT and ODF formulations have five interesting advantages compared to oral formulations if absorption occurs in the mouth (Kathpalia & Gupte, 2013; Cilurzo *et al*., 2018): (1) a shorter T_max_, and T_onset_, depending on drug physicochemical properties and excipients; (2) an increase in drug bioavailability by preventing catabolism of the drug by the stomach and gut, and through the first passage effect; (3) no need to alter drug dosage compared with liquid formulations; (4) discrete intake of the drug since water is not usually required; and (5) elimination of swallowing issues that is present in the general population, specifically in 22% of patients aged > 50 years (Howden, 2004), and more evident in special subpopulations such as geriatric patients and those with dysphagia. The advantages of ODT and ODF formulation and, in particular, points 4 and 5, appear to be crucial for improving patient adherence to treatment. Indeed, the majority of patients prefer ODT/ODF forms over conventional solid oral dosage forms (Dowson & Almqvist, 2005; Jannini & Droupy, 2019).

Technical issues concerning OTDD, ODT, and ODF preparation are beyond the aim of this review, but have been recently presented and discussed in some excellent reviews (Hoffmann *et al*., 2011; Kathpalia & Gupte, 2013; Sattar *et al*., 2014; Cilurzo *et al*., 2018).

## ODT Formulation of Vardenafil

A rapidly disintegrating ODT formulation of vardenafil has been developed by Bayer, and two phase III studies comparing placebo with 10 mg vardenafil ODT, taken on demand, concluded that treatment significantly improves erectile function and is well tolerated in a broad population of men with ED. In the first study, ~55% of men receiving active treatment were > 65 years old (Sperling *et al*., 2010). Treatment was well tolerated, and the main adverse events were headache (16% of patients), flushing (8% of patients), and dyspepsia (4% of patients). In the second study, identical to the first but performed in different countries, the main adverse events were headache (12% of patients), flushing (8% of patients), nasal congestion (5% of patients), and dizziness (3% of patients) (Gittelman *et al*., 2010). An integrated analysis of the two trials concluded that vardenafil ODT significantly improved erectile function in men with ED regardless of age, baseline ED severity, or underlying conditions (Sperling *et al*., 2011).

No head‐to‐head comparison of efficacy and safety has been reported for vardenafil ODT and vardenafil film‐coated tablets, although the pharmacokinetic properties of the two formulations have been compared (Heinig *et al*., 2011). This crossover study demonstrated that vardenafil ODT is absorbed after oral administration without water, with a similar but not identical pharmacokinetic profile to vardenafil film‐coated tablets. The main differences were a bigger AUC and, surprisingly, a longer T_max_ of vardenafil ODT compared with vardenafil film‐coated tablets. In particular, the film‐coated tablet formulation was associated with a sharp vardenafil C_max_ followed by a rapid drop in concentration. By contrast, the vardenafil plasma concentration–time curve for the ODT formulation was characterized by a more plateau‐shaped profile with a lower C_max_ for ODT than film‐coated tablets (81–92% depending on patient age). At the beginning of the absorption phase (20 min, when plasma concentration became measurable), the plasma concentrations of both formulations were similar. Soon after (at 25 min), the concentration of vardenafil ODT began lagging behind that of film‐coated tablets by about 5–10 min. As a result, the median T_max_ of the ODT formulation doubled in young adults (≤45 years, *n* = 14), resulting in values of 1.5 (range = 0.75–2.5) for ODT formulation and 0.75 (range = 0.5–1.5) for film‐coated tablet formulation, after a single dose. Similar results were observed in adults (≤65 years, *n* = 20), resulting in values of 1.25 (range = 0.75–2.5) for ODT formulation and 0.75 (range = 0.5–1.5) for film‐coated tablet formulation, and in old patients (≥65 years, *n* = 14), values were 0.875 (range = 0.5–3.0) for ODT formulation and 0.75 (range = 0.5–3.0) for film‐coated tablet formulation. Thus, ODT vardenafil not only fails to decrease T_max_ in all patients but actually increases it in several patients compared with film‐coated tablet formulation. Moreover, the time taken for vardenafil to reach a hypothetical target plasma concentration is longer for ODT formulation than for film‐coated tablets.

The increase in AUC by 21–44% for ODT formulation compared with film‐coated tablet means that 10 mg ODT is more effective than 10 mg film‐coated tablets, and halfway between 10 and 20 mg for film‐coated tablet formulation. The plateau‐shaped profile of vardenafil ODT may indicate a longer duration for maximal drug effects.

In conclusion, the clinical effects of vardenafil ODT formulation are similar to those of film‐coated tablet formulation (Table 2). Even if ODT formulation increases the bioavailability of vardenafil, its effects and adverse events are similar to those of vardenafil film‐coated tablets, presumably because of the plateau‐shaped profile of the plasma concentration of vardenafil. ODT formulations can be taken even after a meal because the AUC and T_max_ do not change (even if the C_max_ decreases by 35%). Additionally, ODT formulations can be taken without water, therefore improving discretion, but this does not address T_max_/T_onset_ issues.

**Table 2:** Pharmacokinetics of vardenafil formulations in men 45–65 years old (Heinig *et al*., 2011)

	Film‐coated table	ODT formulation
T_max_ (h), median	0.75	1.25
C_max_ (μg/L), geometric mean	8.2	7.2
AUC (μg h/L), geometric mean	23.5	30.0

## ODT, Granule, and Some ODF Formulations of Sildenafil Do Not Shorten T_max_ and T_onset_

A rapidly disintegrating ODT formulation of sildenafil has been developed by Pfizer, and a pharmacokinetic study has been published (Damle *et al*., 2014). In a crossover, single‐dose study, 50 mg of sildenafil was given to 36 healthy subjects (≥45 years) with or without ED as (i) film‐coated tablets (obviously with water), (ii) ODT without water, and (iii) ODT with water. C_max_ and AUC values of sildenafil were comparable across the three treatments. Median T_max_ values were also comparable across treatments, with a median T_max_ of 0.75 h. The effect of food was studied in 12 subjects, and the median T_max_ of sildenafil ODT was 0.63 h (range = 0.25–1.5) when fasting and 4 h (0.5–6.0) when fed with a high‐fat meal. Thus, sildenafil ODT does not appear to be absorbed by the buccal mucosa, but rather by the gastrointestinal system, similar to sildenafil film‐coated tablets.

A fine granular formulation of sildenafil‐free base has been developed by Sam‐A Pharm (Korea), and the pharmacokinetics of this formulation have been studied (Zheng & Kim, 2014). In a crossover, single‐dose study, 50 mg of a film‐coated tablet or granular formulations sildenafil was given to 40 healthy subjects (23–37 years). Median T_max_ values were comparable across treatments, with a median T_max_ of 0.75 h, and C_max_ was slightly higher after taking granule formulation compared with film‐coated tablets (234 vs. 205 ng/mL). The AUC was also slightly higher. These results may suggest that this formulation is also mainly absorbed by the gastrointestinal route.

An ODF formulation of sildenafil has been developed by Seoul Pharma Co. Ltd (Korea). A crossover, single‐dose study describing the pharmacokinetics of this formulation on healthy males (20–50 years) demonstrated that in this case also, the pharmacokinetics of the ODF formulation were very similar to those of film‐coated tablets (Roh *et al*., 2013). The authors tested both 50 mg and 100 mg sildenafil doses when comparing ODF and film‐coated tablet formulations. The AUC and C_max_ values of both doses for the ODF formulation differed by no more than 10% compared with film‐coated tablets, and the median T_max_ of both formulations was identical. The authors evaluated the tolerability of the treatments, and no serious adverse events were reported for either formulation. However, among the 57 patients that received a 100 mg dose of sildenafil, nasal congestion was reported by seven patients receiving the ODF formulation, compared with one patient receiving the film‐coated tablet formulation, and rhinorrhoea was described by five patients (all receiving the ODF formulation). Considering that the incidence of other adverse events (such as headache and abnormal vision) was identical in the two treatment groups, in our opinion a locoregional effect of ODF sildenafil cannot be excluded, possibly suggesting that sildenafil in this ODF formulation was absorbed, at least in part, by the buccal mucosa.

Another ODF sildenafil (50 mg) formulation has been prepared by CL Pharm Co. Ltd. (Asan‐si Chungcheongnam‐do, Korea). A crossover, single‐dose study describing the pharmacokinetics of this formulation on 47 healthy males (mean age of 32 years) was performed recently in Mexico (Aguirre *et al*., 2019). In this study, AUC and C_max_ values for the ODF formulation differed no more than 10% from those of film‐coated tablets. The median T_max_ of the ODF formulation was higher than that of film‐coated tablets (1.25 vs. 0.75). However, one figure in the paper indicates that the shift in the T_max_ of the ODF formulation is not due to a lower absorption rate, but rather to a smoothing of the curve, resulting in a higher plasma concentration of the drug for a longer duration with the ODF formulation. However, a figure of the paper shows that the plasma concentration of the ODF formulation was never higher than that achieved using film‐coated tablets during the absorption phase. In conclusion, consistent with the other studies, a sufficiently high concentration of sildenafil was reached at the same time with both ODF and film‐coated tablet formulations. The authors also evaluated the tolerability of the treatments and found a similar incidence of adverse events with both formulations. However, it is likely that nasal congestion and rhinorrhoea were not evaluated, because the authors do not report patients exhibiting these side effects.

In conclusion, ODT formulation, granule formulation, and both Korean ODF formulations can be taken without water and are thus more discrete, but none decrease the T_max_ or achieve a sufficiently high plasma concentration before film‐coated tablets, leaving the T_max_/T_onset_ issues unresolved (Table 3). The observation of locoregional adverse events with one of the ODF formulations suggests that with this formulation sildenafil is absorbed by the buccal mucosa, at least in part. Therefore, this formulation may decrease the effect of a meal on absorption rate and T_max_. The studies do not confirm the results of the aforementioned work by De Siati *et al*. in which effects of crushed film‐coated tablets were compared with intact film‐coated tablets (De Siati *et al*., 2003). Therefore, it is possible that the observations of these authors are due to the study flaws discussed above.

**Table 3:** Pharmacokinetics of sildenafil formulations (50 mg)

	Film‐coated table. Data from Ref.^a^^,^ b ^,^ c ^,^ d, respectively	ODT formulation from Pfizer^a^ (without water)	Fine granular formulation from Sam‐A Pharm^b^	ODF formulation from Seoul Pharma^c^	ODF formulation from CL Pharm^d^
T_max_ (h), median	0.75, 0.75, 0.75, 0.75	1.00	0.75	0.75	1.25
C_max_ (μg/L), geometric mean	297, 205, 202, 159	272	234	208	150
AUC (μg h/L), geometric mean	846, 621, 489, 398	891	555	514	436

a Damle *et al*. (2014), Asian healthy men 45–69 years old.

b Zheng & Kim (2014), healthy men 23–37 years old.

c Roh *et al*. (2013), Korean healthy men 20–50 years old.

d Aguirre *et al*. (2019), Mexican healthy men 18–55 years old.

## Possible Methods for Increasing the Absorption Rate of Sildenafil in the Mouth

A very interesting study on rabbits evaluated the excipients needed to increase both sildenafil bioavailability and the rate of its absorption by oral mucosa to achieve a rapid onset of action with good efficacy at lower doses (Sheu *et al*., 2016). Sildenafil and its citrate salt were formulated at two dosages in the form of a sublingual spray (five different formulations) and sublingual tablets (20 different formulations). Disintegration time, hardness, and dissolution half‐life of sildenafil sublingual tablets were tested *in vitro*, and several parameters including C_max_, effect duration, T_max_, and T_onset_ (the time needed to achieve 100 nM sildenafil plasma concentration) were evaluated for sprays and sublingual tablets *in vivo*. The authors concluded that most of the sublingual tablets prepared with different binders and disintegrants behaved similarly regarding T_onset_, effect duration, and bioavailability. By contrast, the vehicle played a critical role, both *in vitro* and *in vivo*, and while several achieved rapid sildenafil onset, few achieved high sildenafil bioavailability. In conclusion, sublingual tablets formulated with 0.5 mg sildenafil in propylene glycol adsorbed onto Florite^®^ R at a 1:1 weight ratio then mixed with Cyclocel^®^ and Ac‐Di‐Sol^®^ showed fast onset action (1.9 min) that lasted for ~1 h, with a T_max_ of 67 min and bioavailability of 90%. For comparison, in rabbits, film‐coated tablets containing 50 mg sildenafil showed onset action of 5.5 min that lasted for ~3 h, a T_max_ of 86 min, and bioavailability of 40%. Thus, this study provided proof of principle that some excipients can improve the pharmacokinetics of sildenafil via absorption in the oral mucosa.

## One ODF Formulation of Sildenafil Can Improve T_onset_

In 2004, Deveci *et al*. published a study in which a 20 mg dose of sublingual sildenafil developed by Durus SofTab (Durus Ltd., FL, USA) was tested against placebo in patients with ED in a double‐blind study (Deveci *et al*., 2004). Patients received the drug during sexual stimulation, and they were asked to record the time between receiving the drug and onset of erection. The mean time to achieve a rigid erection was 15.5 min with sublingual sildenafil and 30 min with placebo. Therefore, it may be inferred a short T_onset_. The effect of sublingual sildenafil for completed coitus lasted for an average of 40 min, compared with 20 min for the placebo group. However, the study did not perform a head‐to‐head comparison between sublingual and film‐coated tablets of sildenafil and has some flaws. Firstly, the number of patients studied was low (*n* = 20 in both groups), even considering the wide age range (25–65 years), and the authors did not specify the degree of severity of ED in patients and provide details of the placebo treatment. Moreover, Eardley and colleagues reported that film‐coated tablets of sildenafil yielded a penetrative erection within 12 min in some patients, and within 30 min in most patients (Eardley *et al*., 2002), although the consensus is that oral sildenafil effects are seen after about 1 h, as discussed above. Given that initiation of the effects of sildenafil depends not only on the placebo effect but also on the dose and pharmacokinetics of the formulation, as well as ED severity, only a head‐to‐head study can give some indication of the rapidity by which sildenafil effects are observed following a sildenafil formulation. However, the study encouraged further work to improve the pharmacokinetics of ODT/ODF formulations of sildenafil.

**Figure 3:**
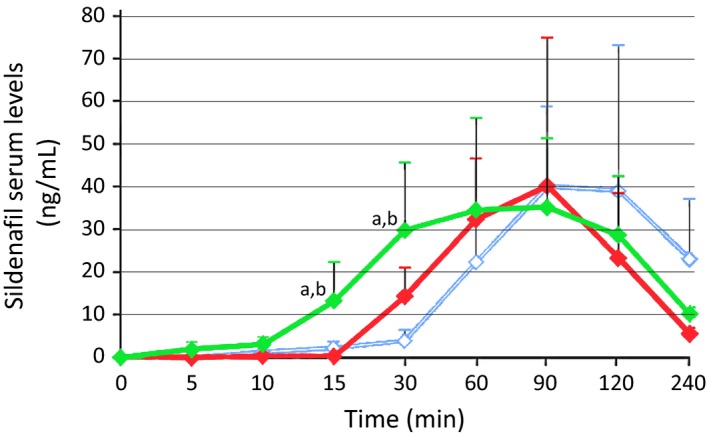
Pharmacokinetic profile of three sildenafil formulations according to De Toni *et al*. (2018). Serum concentrations are shown for 20 ED patients after taking sildenafil ODF (IBSA, green line), sildenafil ODT (Pfizer, red line), and sildenafil film‐coated tablets (Pfizer, light blue line). Modified from Fig. 3 of De Toni *et al*. (2018). ^a^ *p* < 0.01 vs. film‐coated tablets; ^b^ *p* < 0.05 vs. ODT.

Another ODF formulation of sildenafil, developed by IBSA (Switzerland) and marketed in Italy by Sofar and IBSA, is described in three recent studies. De Toni *et al*., (2018) compared the pharmacokinetics of ODF with ODT (Pfizer) and film‐coated tablets (Pfizer) through a crossover, single‐dose study on 20 Italian males with psychogenic ED (mean age of 31 years). Clinicians asked patients to maintain ODT or ODF under the tongue for 15 min to promote the sublingual route. C_max_ was comparable between ODT (46 ng/mL) and film‐coated tablets (49 ng/mL) but was ~22% lower for ODF (38 ng/mL), due to a smoother curve of sildenafil plasma concentration compared with that of film‐coated tablets (Fig. 3). AUC values were comparable between ODF (5898 ng/mL per min) and film‐coated tablets (6180 ng/mL per min), and the value was ~25% lower for ODT (4623 ng/mL per min) than film‐coated tablets. Median T_max_ was not reported by the authors, but mean T_max_ was slightly lower for ODF (70 min) than ODT (90 min) and film‐coated tablets (95 min). More interestingly, at 15 min following ODF intake, the mean sildenafil plasma concentration was > 10 ng/mL; following ODT and film‐coated tablets intake, this was 0 ng/mL, representing a significant difference. Moreover, at 30 min following ODF intake, the mean sildenafil plasma concentration was 30 ng/mL, compared with 15 and 5 ng/mL following ODT and film‐coated tablet intake, respectively (again representing a significant difference). In other words, if we consider 10 ng/mL as the effective plasma concentration of sildenafil, this concentration is reached after 13–14 min following intake of ODF, 25–26 min following intake of ODT, and 40 min following intake of film‐coated tablets. If we consider 20 ng/mL as the effective plasma concentration of sildenafil, this concentration is reached 21–22 min following intake of ODF, 38–40 min following intake of ODT, and 56–58 min following intake of film‐coated tablets. The differences between the three formulations are summarized in Table 4.

**Table 4:** Comparison of the pharmacokinetics of ODF formulations from IBSA with film‐coated tablet formulation and ODT formulation, both from Pfizer (De Toni *et al*., 2018)

	Film‐coated tablet formulation from Pfizer	ODT formulation from Pfizer	ODF formulation from IBSA
C_max_ (ng/mL)	49	46	38
AUC (ng h/mL)	6180	4623	5898
T_max_ (ng/mL), mean^a^	95	90	70
T_onset_ (10 ng/mL)^b^	13.5	25.5	40
T_onset_ (20 ng/mL)^b^	21.5	39	57
Adverse effects			
Nasal congestion	40%	25%	30%
Flushing	50%	20%	20%
Headache	35%	30%	5%

a Non‐fasting patients.

b Derived from a figure of De Toni *et al*. study.

The mean T_max_ of ODT and film‐coated tablets appears to be higher than expected. This may be due to the study protocol which allowed patients to have a non‐fat breakfast (no milk or other fatty food) at least 2 h before the test. This was similar to a real‐life scenario but different from the usual way in which pharmacokinetics of sildenafil is studied (i.e., in fasting men). In our opinion, the differences between ODF and the other formulations in this real‐life scenario further confirm that this ODF sildenafil formulation is absorbed by the mouth, and is therefore not affected by eating.

The same study evaluated the adverse effects of the three formulations (De Toni *et al*., 2018), which were headache (14 patients), flushing (18 patients), and nasal congestion (19 patients). The incidence of the latter was similar for all three formulations (8, 5, and 6 with film‐coated tablets, ODT, and ODF, respectively). Of note, the incidence of headache and flushing was higher with film‐coated tablets and ODT than with ODF (headache = 7, 6, and 1 patients with film‐coated tablets, ODT, and ODF, respectively; flushing = 10, 4, and 4 patients with film‐coated tablets, ODT, and ODF, respectively). These differences may be due to the plateau‐like curve of the plasma concentration of sildenafil following intake of the ODF formulation (compare Fig. 1 and Fig. 3).

Another study evaluated the pharmacokinetics of the above ODF formulations administered by supralingual and sublingual routes to 12 healthy subjects (Loprete *et al*., 2018). The authors found that the sublingual route slightly shortened the T_max_ compared with supralingual ODF, suggesting that the best way to take the ODF formulation is the sublingual route.

A previous study compared film‐coated sildenafil tablets (100 mg) with sildenafil ODF (75 mg) (Cocci *et al*., 2017). The study involved administration of sildenafil film‐coated tablets for 4 weeks, followed by a 2‐week washout period, then administration of sildenafil ODF for 4 weeks. Patients (*n* = 139) were of a mean age of 67 years, and most had mild/moderate ED. The results revealed differences in mean IIEF scores for erectile function, orgasmic function, sexual desire, and intercourse satisfaction that were significantly in favor of sildenafil film‐coated tablets (100 mg), whereas the mean score for overall satisfaction was in favor of sildenafil ODF (75 mg). In our opinion, no conclusion can be drawn from the above study because it has two crucial flaws: (i) In light of pharmacokinetics data supporting a similar AUC value for sildenafil film‐coated tablet and ODF formulations (De Toni *et al*., 2018), comparison of the two formulations at different dosage does not provide useful information, and (ii) when administering a new drug or a new drug formulation, placebo effects can play a relevant role, especially when a reference drug is already used by patients. Therefore, in the above study, the comparison between the formulations should have been carried out using a masked approach (i.e., by administering film‐coated tablets containing placebo plus ODF sildenafil to one group, and film‐coated tablets containing sildenafil plus ODF placebo to the other group). A crossover of patients in the second part of the study would further improve the study. Despite the study flaws, it appears relevant that the mean score for overall satisfaction was in favor of sildenafil ODF (75 mg).

In conclusion, sildenafil ODF prepared in IBSA appears to represent a significant advance in terms of achieving a more rapid absorption of sildenafil. Further head‐to‐head studies are needed to assess the effects and side effects of this new formulation.

## The Excipients that may Improve T_onset_ for ODF from IBSA

Gobry *et al*. (2000) studied the physiochemical properties of sildenafil. According to the authors, sildenafil is a typical ampholyte with modest basicity (pKa1 = 6.78) and weak acidity (pKa2 = 9.12). Therefore, the compound is mostly neutral at physiological pH and has relatively high lipophilicity. This may favor rapid adsorption via the buccal mucosa. However, a drug must also have a certain degree of hydrophilicity to dissolve in the saliva to reach the mucosa (Rathbone *et al*., 2004).

Wang *et al*. (2008) studied the overall solubility and permeability of sildenafil across artificial membranes, and they reached some different conclusions from Gobry *et al*. but agreed that the solubility of sildenafil in water is very low, and this is a limiting factor for a good absorption via the buccal mucosa. Therefore, to improve the efficiency of sublingual absorption, the solubility of sildenafil in saliva should be maximized, while retaining sufficient lipophilicity. Wang *et al*. suggested that the two optimum pH values are 4.50 and 10.24, where the solubility of either cationic or neutral species (pH 4.5) or anionic and neutral species (pH 10.24) is maximal, and maximal transmucosal flux may be achieved.

In conclusion, these studies suggest that sildenafil is a complex molecule and ensuring contact with the buccal mucosa is not sufficient to promote adsorption. Therefore, transmucosal delivery can be achieved only with specific excipients.

Presently, the reason why the different ODT, granule, and ODF formulations have different T_max_ and T_onset_ is unknown. We researched the excipients present in the ODF formulation, the ODT formulation, and the film‐coated tablet formulation (Table 5) that are the three formulations studied by De Toni *et al*., (2018). As film‐coated tablets are not done to be crushed in the mouth, the excipients are different from those of ODT and ODF formulations, as shown in Table 5. Although ODT and ODF formulations are technically different, requiring different excipients, the analyzed ODT and ODF formulations share some excipients, including polyvinyl acetate, a diluent, and film‐forming ingredient. Moreover, maltodextrin is cited as the major excipient in the ODF formulation and is a carrier usually presents in large amounts (30–50%) in ODF formulations (Parikh *et al*., 2014). Presently, whether maltodextrin plays a role in shortening the T_onset_ of ODF is difficult to say.

**Table 5:** Excipients in film‐coated tablet formulation from Pfizer, ODT formulation from Pfizer, and ODF formulation from IBSA as reported in the respective data sheets

	Film‐coated tablet formulation from Pfizer^a^	ODT formulation from Pfizer^b^	ODF formulation from IBSA^c^^,^ d
Excipients	Microcrystalline cellulose, calcium hydrogen phosphate (anhydrous), croscarmellose sodium, magnesium stearate (only the tablet core)	Mannitol, crospovidone, polyvinyl acetate, povidone (the four ingredients of the diluent), croscarmellose sodium (disintegrant), microcrystalline cellulose (filler), silica colloidal anhydrous (glidant), sucralose (sweetener), indigo carmine aluminum lake E132 (colorant), magnesium stearate (lubricant), maltodextrin, dextrin, propylene glycol, glycerol, alpha‐tocopherol and flavoring ingredients (the latter six ingredients are part of commercially available sweeteners and flavors)	Maltodextrin, glycerol, polysorbate 20, monocaprylate of propylene glycol, polyvinyl acetate in dispersion (30%), lemon and grapefruit flavoring (essential oil of lemon, citrate, linalool, essential oil of grapefruit, essential oil of orange, nootkatone, butylated hydroxyanisole E320, ascorbic acid E300, maltodextrin, Arabic gum E414), sucralose, titanium dioxide, indigo carmine

a Formulation obtained from datasheet queried on January 2019 and available at the URL: https://www.ema.europa.eu/documents/product-information/viagra-epar-product-information_en.pdf.

b Formulation obtained from datasheet queried on January 2019 and available at the URL: https://www.ema.europa.eu/documents/variation-report/viagra-h-c-202-x-0070-epar-assessment-report-extension_en.pdf.

c Formulation obtained from datasheet queried on January 2019 and available at the URL: https://farmaci.agenziafarmaco.gov.it/aifa/servlet/PdfDownloadServlet?pdfFileName=footer_000299_044358_FI.pdf&retry=0&sys=m0b1l3.

d Translated from the Italian data sheet.

Of note, in the ODF formulation, a surfactant (polysorbate 20, also known as Tween 20) and a cosurfactant (monocaprylate of propylene glycol) are present. Surfactants act as a solubilizing/dispersing agent to ensure that films are dissolved within seconds and the active agent is released quickly (Siddiqui *et al*., 2011). It is reasonable to assume that the use of both excipients may help to increase the adsorption rate of sildenafil, but specific studies are needed.

In our opinion, another interesting excipient present exclusively in the ODF formulation is ascorbic acid. This molecule works in two ways: as a saliva‐stimulating agent and a pH‐lowering molecule. Saliva‐stimulating agents increase the rate of production of saliva and increase the amount of drug dissolved, especially drugs such as sildenafil with low solubility in water (Kathpalia & Gupte, 2013). Ascorbic acid has a pH of ~2.5 when dissolved in deionized water. The pH of saliva in the presence of ascorbic acid is likely to be higher, depending on the buffering potential of saliva (Hara & Zero, 2014) and the concentration of ascorbic acid. Although experimental data are not available, the pH of saliva under the tongue in contact with ODF releasing ascorbic acid (and in which sildenafil must dissolve) is likely to be ~4–5. This is close to the pH of 4.5 at which maximal transmucosal flux of sildenafil may be achieved, according to Wang *et al*., (2008). Whether ascorbic acid plays a role in shortening T_onset_ of ODF formulation has to be determined.

In conclusion, specific surfactants, cosurfactants, and ascorbic acid may be the key excipients shortening the T_onset_ and T_max_ of the ODF formulation from IBSA compared with ODT from Pfizer. However, studies on the roles of these excipients in experimental models are needed. In fact, it can be hypothesized that different concentrations of these and other excipients may even improve the oral transmucosal absorption.

## Conclusions

Rapid onset of the effects of PDE5is is a requirement when PDE5is are taken on demand. Most of the sildenafil formulations show too long T_onset_ in fasting patients. In real life, patients tend to engage in sexual activity after an evening meal, which further slows the onset of the effects. Rapid onset of the effects of PDE5is can eliminate the need for planning intercourse, thereby increasing the spontaneity of sex and assisting the desire of both partners, and can increase the likelihood of a completely hard and fully rigid erection, resulting in increased sexual satisfaction. This could also improve the placebo effect connected with drug intake. To confirm that the rapid onset of the PDE5i effect plays a role in increasing patient satisfaction and decreasing treatment dropouts, head‐to‐head studies with blinded drug administration need to be performed.

It is likely that oral transmucosal drug delivery achieves a rapid onset of PDE5i effects, regardless of empty stomach. Moreover, this form allows greater discretion, as it does not require the intake of water. However, several ODT and ODF formulations of sildenafil exhibit pharmacokinetics that are very similar to those of film‐coated tablets, probably because of the use of inappropriate excipients required by the peculiar physiochemical properties of sildenafil. Some evidence suggests that these formulations are mainly absorbed by the gastrointestinal route.

The pharmacokinetic profile of a more recent ODF formulation indicates rapid absorption via the oral mucosa, which likely achieves a more rapid onset of the sildenafil effects. However, head‐to‐head studies with blinded drug administration in patients before and after regular meals are needed.

## Conflict of Interest

Manuscript writing and editing were supported by SOFAR. However, SOFAR did not participate in study conception and manuscript writing. Giuseppe Nocentini is a consultant of Pieris Pharmaceuticals and Amgen. The other authors have no conflicts of interest.

## Authors’ Contributions

A. Zucchi and G. Nocentini orchestrated the review and determined its structure. G. Nocentini wrote the review with suggestions and contributions from all authors. L. Cari prepared the Figures. L. Cari and M.G. Petrillo prepared the Tables.
